# Flavonoids in *Ageratum conyzoides* L. Exert Potent Antitumor Effects on Human Cervical Adenocarcinoma HeLa Cells *In Vitro* and *In Vivo*

**DOI:** 10.1155/2020/2696350

**Published:** 2020-05-04

**Authors:** Zeyan Lin, Yanyan Lin, Jinxing Shen, Meijiao Jiang, Youming Hou

**Affiliations:** ^1^Department of Pharmacy, Zhangzhou Health Vocational College, Zhangzhou, 363000 Fujian, China; ^2^Faculty of Basic Medicine, Zhangzhou Health Vocational College, Zhangzhou, 363000 Fujian, China; ^3^Biobank, The First Affiliated Hospital of Xiamen University, Xiamen, 361003 Fujian Province, China; ^4^Zhangzhou Hospital of Fujian Medical University, Zhangzhou, 363000 Fujian, China; ^5^State Key Laboratory of Ecological Pest Control for Fujian and Taiwan Crops, Fujian Agriculture and Forestry University, Fuzhou 350002, China; ^6^Fujian Provincial Key Laboratory of Insect Ecology, Department of Plant Protection, Fujian Agriculture and Forestry University, Fuzhou, Fujian, China

## Abstract

The *Ageratum conyzoides* L. (*A. conyzoides*) is commonly used as a traditional medicine, and its antitumor effects have also been studied. However, the functional roles of flavonoids in *A. conyzoides* in antitumor activities have not been clarified. The present study is aimed at investigating the biological effects of flavonoids in *A. conyzoides* on human cervical adenocarcinoma. Firstly, we detected that flavonoids in *A. conyzoides* significantly inhibited the proliferation, invasion, migration, and clonality of human cervical adenocarcinoma HeLa cells *in vitro*. Furthermore, we found that flavonoids in *A. conyzoides* induced significant S phase arrest and apoptosis and obviously decreased the intracellular reactive oxygen species (ROS) level in HeLa cells. Finally, we found that flavonoids in *A. conyzoides* significantly inhibited the HeLa xenograft tumor growth and epithelial-mesenchymal transition (EMT) *in vivo*. In conclusion, our results demonstrated the obvious antitumor effects of flavonoids in *A. conyzoides* on HeLa cells, suggesting that flavonoids in *A. conyzoides* could be provided as a novel therapeutic compound for human cervical adenocarcinoma.

## 1. Introduction

Cervical cancer is the fourth most common cancer in women worldwide [1, 2] and accounts for approximately 275,000 deaths and 528,000 diagnosed cases per year [3]. The main cause for cervical cancer is infection with the human papilloma virus (HPV), and the most common subtype of HPV in cervical cancer is HPV 16, followed by HPV 18, HPV 45, and HPV 31 [4, 5]. Current therapies for cervical cancer including surgery, chemotherapy, or radiotherapy are expensive, nonspecific, and low effective [6]. In addition, several unsatisfactory problems in tumor chemotherapy are the intrinsic or acquired drug resistance of tumor cells as well as high toxic side effects of chemotherapeutic drugs [7]. Hence, it is creditable to exploit more novel therapeutic compounds for cervical cancer treatment.

Nowadays, natural products are considered attractive candidates for new tumor therapies ascribed to their properties of chemical diversity, structural complexity, affordability, few toxic effects, and inherent biological activities [8]. Flavonoids are a large class of natural compounds that are widely found in the plant kingdom [9]. Flavonoids possess a diverse range of bioactivities, such as antioxidation, antihyperlipidemia, antifatigue, antiaging, and atherosclerosis-prevention activities [10]. Meanwhile, the antitumor effects of flavonoids have also been revealed by plentiful of studies [11–13].

*A. conyzoides* is an annual herb which belongs to the Asteraceae family and is generally used as a traditional medicine [14]. It has been reported that *A. conyzoides* exhibited considerable cytotoxic activity on human non-small-cell lung cancer and mouse leukemia cells [15]. To date, more than 21 flavonoids have been extracted from essential oil of *A. conyzoides* [16], and their functional roles in antitumor activities have not been clarified. In the current study, we demonstrated the significant inhibitory effects of flavonoids in *A. conyzoides* on human cervical adenocarcinoma HeLa cells *in vivo* and *in vitro*.

## 2. Materials and Methods

### 2.1. Cell Culture

The human cervical adenocarcinoma HeLa cells were purchased from the School of Life Sciences, Xiamen University. Cells were cultured in Dulbecco's modified Eagle medium (DMEM, HyClone, Logan, USA), supplemented with 10% fetal bovine serum (FBS, Gibco Life Technologies, NY, USA), and maintained at 37°C in a 95% O_2_ and 5% CO_2_ incubator.

### 2.2. MTT Cell Viability Assay

HeLa cells were seeded into a 96-well culture plate at a density of 500 cells/well and allowed to grow in DMEM supplemented with 10% FBS for 24 h before treatments. Thereafter, cells in three reduplicative wells were incubated with increasing concentrations (50, 100, 200, 300, 400, and 500 *μ*g/mL) of flavonoids in *A. conyzoides* (purchased from the herbal garden of Zhangzhou Health Vocational College, Fujian, China) for 16 h. Cells incubated with sterile ddH_2_O were considered the control. For the MTT assay, medium in each well was carefully replaced by 150 *μ*L fresh DMEM+10% FBS with diluted MTT (0.2 mg/mL, Amresco, USA) and incubated for 4 h at 37°C. Afterwards, the incubation medium was removed and formazan crystals were dissolved in 150 *μ*L solution of DMSO in each well. The OD value of each well was quantified by recording the light absorbance at 630 nm using a microplate reader (Bio-Rad, Hercules, USA). The calculation equation of cell survival rate is as follows: cell survival rate (%) = OD_flavonoids_/OD_control_ × 100%.

### 2.3. Wound Healing Assay

HeLa cells were seeded into the 6-well culture plate and cultured in DMEM without serum until 95% confluence. A wound was then scratched into the cell monolayer using a sterile 10 *μ*L pipette tip. After carefully removing the floating cells with sterile PBS, the remaining adherent cells were cultured at 37°C for 48 h in FBS-free DMEM dissolved with various concentrations of flavonoids in *A. conyzoides* (50, 200, and 400 *μ*g/mL) or sterile ddH_2_O (control). The wound areas were photographed at the 0 h and 48 h time points under an inverted optical microscope-camera system (CK40, Olympus, Tokyo, Japan). The experiments were conducted in triplicate.

### 2.4. Transwell Assay

The 24-well transwell chambers with 8 *μ*m pores (Millipore, Billerica, USA) were used for transwell assays. HeLa cells were seeded into the upper chambers at a density of 1 × 10^5^ and incubated with FBS-free DMEM dissolved with various concentrations of flavonoids in *A. conyzoides* (50, 200, and 400 *μ*g/mL) or sterile ddH_2_O (control). The lower chambers contained the DMEM supplemented with 10% FBS. After incubation for 48 h, cells on the internal surface of the upper chambers were washed with PBS, fixed with 4% paraformaldehyde for 20 min, stained with 0.1% crystal violet (Beyotime, Shanghai, China) for 20 min, and then rinsed with PBS for three times. Finally, three random views for each chamber were captured using an inverted optical microscope-camera system (Olympus), and the number of migration cells in each view was counted. Three independent experiments were performed.

### 2.5. Clonogenic Assay

HeLa cells were plated into culture dishes at a density of 1000 cells/dish and cultured in DMEM supplemented with 10% FBS overnight. Subsequently, various doses of flavonoids in *A. conyzoides* (50, 200, and 400 *μ*g/mL) or sterile ddH_2_O (control) were added into the culture dishes, respectively. The culture medium was replaced every 7 days. After culturing for 14 days, the colonies were fixed with 4% formaldehyde for 20 min, stained with 0.1% crystal violet for 20 min, and then washed with PBS thrice. Finally, the number of colonies was counted. The experiments were performed in triplicate.

### 2.6. Flow Cytometric Analysis

HeLa cells were cultured in full medium dissolved with various doses of flavonoids in *A. conyzoides* (50, 100, 200, 300, and 400 *μ*g/mL) or sterile ddH_2_O (control) for 24 h. For cell cycle analysis, HeLa cells were then collected and fixed with 70% ethanol overnight at 4°C, washed with PBS, and incubated with propidium iodide (PI, Sangon Biotech Co., Shanghai, China) for 10 min. For apoptosis analysis, HeLa cells were harvested and incubated with Annexin-V-fluorescein isothiocyanate (FITC)/PI (Sangon Biotech Co.) at room temperature in the dark for 15 min. Analyses were performed with a flow cytometer (Beckman Coulter, Miami, USA). Each experiment was repeated three times.

### 2.7. Detection of Intracellular Reactive Oxygen Species (ROS)

HeLa cells were seeded into 6-well plates and treated with various doses of flavonoids in *A. conyzoides* (50, 100, 200, 300, and 400 *μ*g/mL) or sterile ddH_2_O (control) for 24 h. Cells were then harvested, washed with PBS, and centrifuged at 1000 rpm for 5 min. After abandoning the supernatant, cells were resuspended and incubated with 2′,7′-dichlorofluorescin diacetate solution (10 *μ*M, Sigma, St Louis, USA) at 37°C for 30 min. Subsequently, cells were washed with PBS and acquired by a flow cytometer (Beckman Coulter) to detect intracellular ROS. The experiments were conducted in triplicate.

### 2.8. Acridine Orange/Ethidium Bromide (AO/EB) Staining

HeLa cells were plated onto the sterile glass coverslips and cultured in DMEM supplemented with 10% FBS. After culturing for 24 h, cells were treated with various doses of flavonoids in *A. conyzoides* (50, 200, and 400 *μ*g/mL) or sterile ddH_2_O (control) for 24 h. Cells were then washed with PBS and stained with 100 *μ*g/mL AO and 100 *μ*g/mL EB (Amresco) in the dark. Finally, cells were viewed and photographed using a fluorescent microscope (Olympus). Cells with bright green fluorescence and orange-red fluorescence in the pyknotic nuclei were considered the early and late apoptotic cells, respectively. The experiments were performed in triplicate.

### 2.9. Xenograft Tumor Assay

All animal experiments were performed in accordance with the National Institutes of Health Guide for the Care and Use of Laboratory Animals and were approved by the Institutional Animal Care and Use Committee of Fujian Agriculture and Forestry University. Four-week-old female nude mice were purchased from Vital River Laboratory Animal Technology Co. (Beijing, China) and randomly divided into the control and experimental groups (4 mice per group). HeLa cells (1 × 10^6^) were subcutaneously injected into the back of nude mice. After injection for one week, nude mice were daily received intraperitoneal injections of flavonoids in *A. conyzoides* (400 mg/kg body weight) or sterile ddH_2_O (control). All nude mice were sacrificed by overdose of urethane at the 4th week, and the tumor weights were measured.

### 2.10. Immunohistochemical (IHC) Staining

The xenograft tumors were fixed with 4% formaldehyde, embedded in paraffin, and cut into 4 *μ*m sections. After incubating with EDTA antigen retrieval solution (Beyotime) at 95°C for 15 min, the slides were incubated with endogenous peroxidase blocking solution (Beyotime) at room temperature for 10 min and then were incubated at 4°C overnight with the primary antibodies: anti-E-cadherin, anti-N-cadherin, and anti-vimentin (ab76055, ab76011, and ab92547; Abcam, Cambridge, UK). Thereafter, the slides were incubated with the corresponding secondary antibody (Beyotime) for 30 min at 37°C and orderly stained with diaminobenzidine (Beyotime) and hematoxylin. Finally, the slides were visualized and photographed under an inverted optical microscope-camera system (Olympus).

### 2.11. Statistical Analysis

Data are presented as the mean ± SD. Statistical analysis was performed with SPSS version 19.0 (SPSS Inc., Chicago, USA). Significant difference was analyzed using one-way analysis of variance (ANOVA), and *p* < 0.05 was considered statistically significant.

## 3. Results

### 3.1. Flavonoids in *A. conyzoides* Inhibited the Proliferation, Invasion, Migration, and Clonality of HeLa Cells *In Vitro*

**Figure 1:**
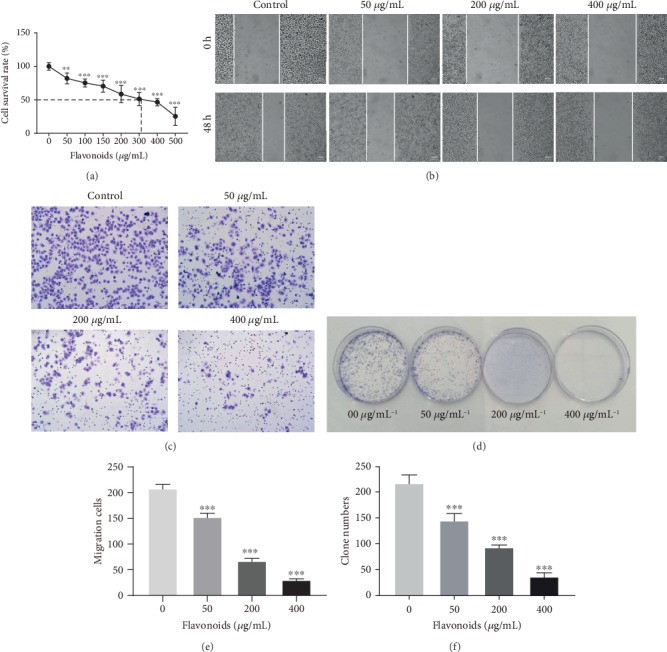
Flavonoids in *A. conyzoides* inhibited the proliferation, invasion, migration, and clonality of HeLa cells *in vitro*. (a) Effects of gradient concentrations of flavonoids (50-500 *μ*g/mL) on the cell survival rate of HeLa cells were tested using the MTT assay (^∗∗^*P* < 0.01 and ^∗∗∗^*P* < 0.001, compared to control). After treatments with increasing doses of flavonoids (50, 200, and 400 *μ*g/mL) for 48 h, the invasion ability of HeLa cells was tested by the wound healing assay (b), and the migration ability (c, e) and clonality (d, f) of HeLa cells were investigated using the transwell and clonogenic assays, respectively (^∗∗∗^*P* < 0.001, compared to control).

Using the MTT assay, we found that flavonoids significantly suppressed the cell survival rate of HeLa cells in a concentration-dependent manner, and the calculated IC_50_ value was 334.171 *μ*g/mL (Figure 1(a)). Next, we detected that the medium (200 *μ*g/mL) and high (400 *μ*g/mL) concentrations of flavonoids obviously reduced the invasion ability of HeLa cells compared to the control (0 *μ*g/mL) and low concentration (50 *μ*g/mL) in the wound healing assay (Figure 1(b)). Furthermore, upon the administration of increasing doses of flavonoids (50, 200, and 400 *μ*g/mL), we found that the number of migration cells and clones was significantly decreased in the transwell and clonogenic assays of HeLa cells, and flavonoids at the concentration of 400 *μ*g/mL possessed the most prominent inhibitory effects (Figures 1(c)–1(f)). Taken together, we suggested that flavonoids in *A. conyzoides* significantly inhibited the proliferation, invasion, migration, and clonality of HeLa cells.

### 3.2. Flavonoids in *A. conyzoides* Induced Significant S Phase Arrest and Apoptosis in HeLa Cells

**Figure 2:**
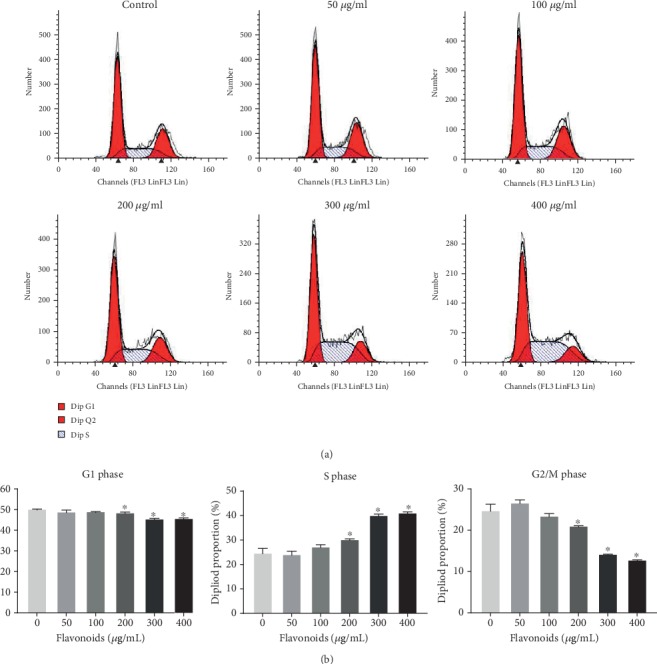
Flavonoids in *A. conyzoides* induced significant S phase arrest in HeLa cells. (a) Cell cycle analyses in HeLa cells upon the administration of increasing doses of flavonoids (50, 100, 200, 300, and 400 *μ*g/mL) using a flow cytometer. (b) Statistical analyses of the proportion of HeLa cells during G1, S, and G2/M phases, respectively (^∗^*P* < 0.05, compared to control).

After being treated with various doses of flavonoids (0, 50, 100, 200, 300, and 400 *μ*g/mL), the cell cycle of HeLa cells was tested using a flow cytometer (Figure 2(a)). We found that flavonoids at low concentrations (50 and 100 *μ*g/mL) had no significant impacts on the cell cycle of HeLa cells. While we detected that flavonoids at concentrations of 200, 300, and 400 *μ*g/mL mildly reduced the proportion of cells in G1 phase and significantly increased and decreased the proportion of cells in S phase and G2/M phase, respectively (Figure 2(b)). These data indicated that flavonoids in *A. conyzoides* could induce significant S phase arrest in HeLa cells.

**Figure 3:**
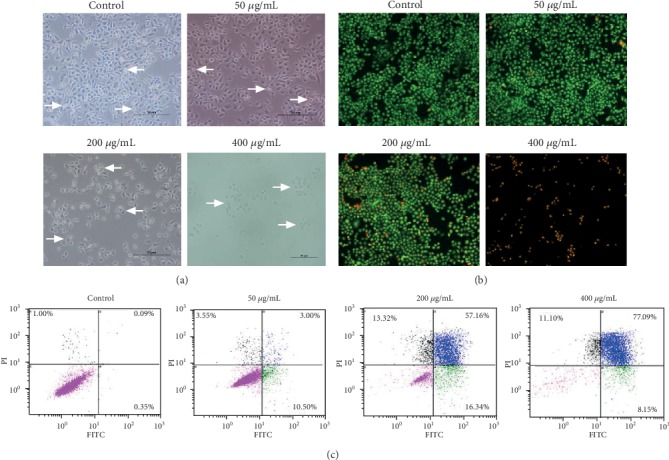
Flavonoids in *A. conyzoides* induced significant apoptosis in HeLa cells. (a) Influences of increasing doses of flavonoids (50, 200, and 400 *μ*g/mL) on the common morphology and density of HeLa cells were observed under the light microscope. White arrows point to the round cells (apoptosis cells). (b) Apoptosis in HeLa cells after treatments with increasing doses of flavonoids (50, 200, and 400 *μ*g/mL) was detected by AO/EB staining. Cells with light green fluorescence in the homogeneous nuclei were considered the survival cells. Cells with bright green fluorescence and orange-red fluorescence in the pyknotic nuclei were considered the early and late apoptotic cells, respectively. (c) Apoptotic analyses in HeLa cells after treatments with increasing doses of flavonoids (50, 200, and 400 *μ*g/mL) using a flow cytometry (black spots: necrotic cells, blue spots: late apoptotic cells, green spots: early apoptotic cells, and pink spots: survival cells).

We further tested that whether flavonoids in *A. conyzoides* could induce apoptosis in HeLa cells. Under the light microscope, we found that HeLa cells without flavonoid treatment were spindle-shaped with a plump body, and there were a small number of round cells scattered in a high-power field (HPF). The low concentration (50 *μ*g/mL) of flavonoids showed no obvious influences on the morphology of HeLa cells. The medium concentration (200 *μ*g/mL) of flavonoids obviously increased the amount of round cells in a HPF and changed the morphology of some HeLa cells to irregular polygons. After treatment with flavonoids at a concentration of 400 *μ*g/mL, almost all HeLa cells became round cells, and the cell density was dramatically decreased in a HPF (Figure 3(a)). Furthermore, we found that HeLa cells without or with low concentration (50 *μ*g/mL) of flavonoid treatment emitted light green fluorescence in the homogeneous nuclei after AO/EB staining. After treatment with flavonoids at a concentration of 200 *μg*/mL, the amount of HeLa cells in a HPF that displayed bright green or orange-red fluorescence in the pyknotic nuclei was obviously increased. After treatment with flavonoids at a concentration of 400 *μ*g/mL, the large majority of HeLa cells in a HPF emitted orange-red in the pyknotic nuclei (Figure 3(b)). These results indicated that the number of apoptotic HeLa cells was significantly increased by flavonoids. Finally, we confirmed that the proportion of apoptotic HeLa cells was significantly elevated by increasing doses of flavonoids (50, 200, and 400 *μ*g/mL) using the flow cytometric analysis. Meanwhile, we found that flavonoids (200, 400 *μ*g/mL) mainly increased the proportion of late apoptotic HeLa cells compared to the control (Figure 3(c)).

### 3.3. Flavonoids in *A. conyzoides* Obviously Reduced the Intracellular ROS Level in HeLa Cells

**Figure 4:**
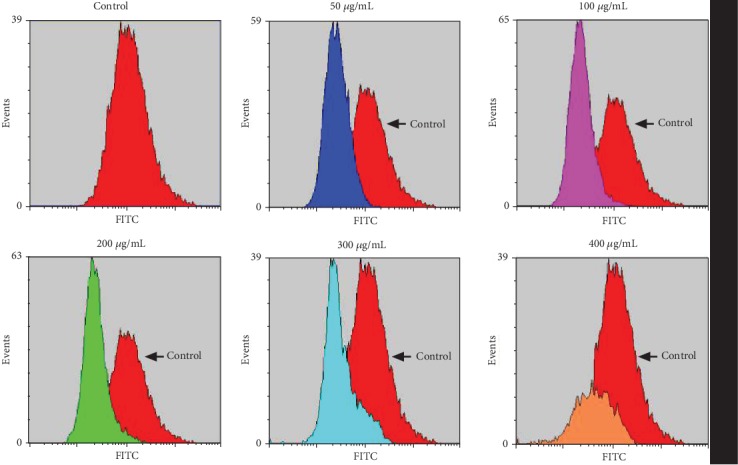
Flavonoids in *A. conyzoides* obviously reduced the intracellular ROS level in HeLa cells. Intracellular ROS levels in HeLa cells upon the administration of increasing doses of flavonoids (50, 100, 200, 300, and 400 *μ*g/mL) were detected using a flow cytometer. Black arrows point the ROS peak of the control group.

We further detected the influences of flavonoids in *A. conyzoides* on the intracellular ROS level in HeLa cells using a flow cytometer. We found that all the ROS peaks in HeLa cells after treatments with various doses of flavonoids (50, 100, 200, 300, and 400 *μ*g/mL) were shifted to the left compared to that of the control group (Figure 4), indicating that the intracellular ROS level in HeLa cells was reduced by flavonoids in *A. conyzoides*.

### 3.4. Flavonoids in *A. conyzoides* Significantly Suppressed the HeLa Xenograft Tumor Growth and EMT *In Vivo*

**Figure 5:**
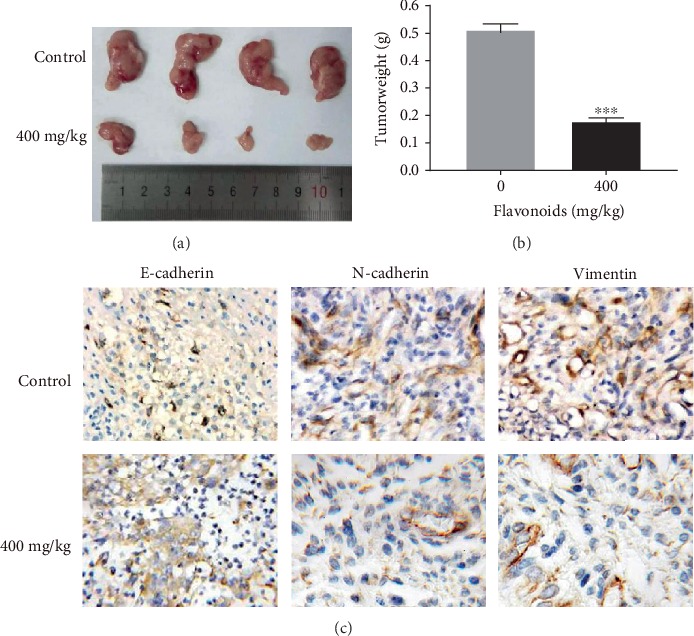
Flavonoids in *A. conyzoides* significantly suppressed the HeLa xenograft tumor growth and EMT *in vivo*. (a, b) Effects of flavonoids (400 mg/kg) on the volume and weight of HeLa xenograft tumor (^∗∗∗^*P* < 0.001, compared to control (sterile ddH_2_O)). (c) Expressions of E-cadherin (epithelial marker), N-cadherin, and vimentin (mesenchymal markers) in the xenograft tumors were detected using IHC staining.

In xenograft tumor assay, we found that no death occurred in all the experimental nude mice with daily injection of flavonoids in *A. conyzoides* (400 mg/kg) for three weeks. We detected that flavonoids (400 mg/kg) significantly suppressed the growth of HeLa cells compared to the control (Figure 5(a)). The weight of xenograft tumors treated by 400 mg/kg flavonoids was significantly higher than that of the control group (Figure 5(b)). Moreover, using the IHC staining, we found that flavonoids (400 mg/kg) prominently enhanced the E-cadherin expression and reduced the N-cadherin and vimentin expressions in the xenograft tumors (Figure 5(c)), indicating that flavonoids in *A. conyzoides* could inhibit the EMT *in vivo*.

## 4. Discussion

A previous study has reported that essential oils of A. conyzoides could significantly inhibit the proliferation of prostate cancer cell lines (LNCaP and PC-3) and glioblastoma cell lines (SF-763 and SF-767) [17]. Another research has also proven that several flavonoids isolated from the Asteraceae family of plants showed considerable suppressive effects on the growth of rat glial tumor cells (C6) [18]. Consistently, our current study demonstrated that flavonoids *in A. conyzoides* significantly inhibited the growth of human cervical adenocarcinoma HeLa cells *in vitro* and *in vivo*, and the calculated IC_50_ value was 334.171 *μ*g/mL. It has been reported that chronic treatment with the ethanolic leaf extract of *A. conyzoides* at 200, 400, and 600 mg/kg body weight did not significantly change the alanine aminotransferase (ALT) and aspartate aminotransferase (AST) expression levels in serum and liver of all experimental animals [19]. In our study, we also found that daily injection of flavonoids in *A. conyzoides* at 400 mg/kg body weight for three weeks did not cause death in all the experimental nude mice. Taken together, we suggest that flavonoids in *A. conyzoides* could be served as a novel and safe therapeutic compound for human cervical adenocarcinoma.

To our knowledge, the cell cycle is divided into four phases: G1, G2, S, and M; and each phase is found to possess its own biological function [20]. Numerous studies have clarified that flavonoids could prominently affect the cell cycle in various cancer cells. For instance, it has been reported that various kinds of flavonoids could induce G0/G1 phase or G2/M phase arrest in breast cancer cells [21]. Zhang et al. have proven that flavonoids extracted from Chinese bayberry leaves could significantly induce G1 cell cycle arrest in ovarian cancer cells [22]. Nagappan et al. have reported that flavonoids isolated from Citrus platymamma induced G2/M cell cycle arrest in A549 human lung cancer cells [23]. In our present study, we found that flavonoids in *A. conyzoides* induced significant S phase arrest in HeLa cells. These findings indicate that various kinds of flavonoids could exert their antiproliferation effects on diverse cancer cells via inducing different cell cycle arrests.

A number of studies have revealed that flavonoids could induce apoptosis in various cancer cells [24–26]. In the present study, we also detected obvious apoptosis in HeLa cells upon the administration of flavonoids in *A. conyzoides*. We think that the round cells increased by flavonoids in *A. conyzoides* in a HPF under the light microscope were apoptotic cells. To confirm this, we performed AO/EB staining, in which we found that the medium concentration of flavonoids in *A. conyzoides* slightly increased the number of early apoptotic HeLa cells (bright green fluorescence), and the medium and high concentrations of flavonoids in *A. conyzoides* both remarkably increased the amount of late apoptotic HeLa cells (orange-red fluorescence). Finally, using the flow cytometric analysis, we further confirmed that the medium and high concentrations of flavonoids significantly increased the percentage of late apoptotic HeLa cells compared to the control. These results indicate that flavonoids in *A. conyzoides* mainly induce late apoptosis in HeLa cells.

Moreover, several studies have reported that flavonoids could induce apoptosis in the bladder and hepatocellular cancer cells via enhancing the intracellular ROS level [27, 28]. On the contrary, we tested that the intracellular ROS levels in HeLa cells were obviously reduced after treatment with increasing doses of flavonoids in *A. conyzoides* in our study. This is consistent with a previous report that dihydromyricetin, a kind of flavonoids extracted from *Ampelopsis grossedentata*, could reduce ROS accumulation in human hepatoma HepG2 cells in a concentration-dependent manner [29]. Fan et al. have also reported that luteoloside, a flavone subclass of flavonoids, could significantly decrease the intracellular ROS level in human hepatoma Huh-7 and SMMC-7721 cells [30]. Based on the above findings, we suggest that flavonoids in *A. conyzoides* could induce apoptosis in HeLa cells through a ROS-independent mechanism.

EMT is a biological process during which epithelial cells lose their epithelial-like phenotypes and acquire the mesenchymal-like phenotypes, which include enhanced migratory potential and invasiveness [31]. It is typically characterized by the coordinated gain and loss of the mesenchymal (e.g., N-cadherin and vimentin) and epithelial (e.g., E-cadherin) markers, respectively [32]. Fan et al. have showed that two dietary flavonoids luteolin and quercetin could inhibit the EMT in squamous carcinoma cells [33]. Chen et al. have also reported that isoliquiritigenin, one of the flavonoids in licorice, significantly inhibited ovarian cancer metastasis through the suppression of EMT [34]. Consistently, in the current study, we found that flavonoids in *A. conyzoides* obviously increased the E-cadherin expression and decreased the N-cadherin and vimentin expressions in the xenograft tumors, which indicated the inhibition of EMT. Hence, we suggest that flavonoids in *A. conyzoides* could significantly inhibit the invasion and migration of HeLa cells via suppressing the EMT. Ascribed to the cells during which EMT could gain the resistance to apoptosis [31], we speculate that the EMT inhibition may be also involved in the apoptosis process of HeLa cells induced by flavonoids in *A. conyzoides*.

A limitation of the present study is that we only preliminarily investigated the antitumor effects of flavonoids in *A. conyzoides* on the human cervical adenocarcinoma HeLa cells, while cervical squamous cell carcinoma is the most common histological type of cervical cancer worldwide [35]. Therefore, the impacts of flavonoids in *A. conyzoides* on other human cervical squamous cell carcinoma cell lines should be also clarified to further confirm their antitumor effects on the human cervical squamous cell carcinoma. Furthermore, the possible mechanism underlying the flavonoids in *A. conyzoides* inducing apoptosis in HeLa cells should also be explored in the further researches.

## 5. Conclusions

In conclusion, the present study demonstrated that flavonoids in *A. conyzoides* significantly inhibited the growth of HeLa cells *in vitro* and *in vivo* via inducing S cell cycle arrest and apoptosis. Moreover, we detected that flavonoids in *A. conyzoides* obviously decreased the intracellular ROS level in HeLa cells. Finally, we clarified that flavonoids in *A. conyzoides* significantly inhibited the invasion and migration of HeLa cells probably through suppressing the EMT. Our findings indicate that flavonoids in *A. conyzoides* could be provided as a novel therapeutic compound for human cervical adenocarcinoma.

## Data Availability

The data used to support the findings of this study are available from the corresponding author upon request.

## Abbreviations

*A. conyzoides*: *Ageratum conyzoides* L.
AO/EB: Acridine orange/ethidium bromide
ALT: Alanine aminotransferase
AST: Aspartate aminotransferase
DMEM: Dulbecco's modified Eagle medium
EMT: Epithelial-mesenchymal transition
FBS: Fetal bovine serum
IHC: Immunohistochemical
PI: Propidium iodide
ROS: Reactive oxygen species.
